# Spatial distribution and determinants of tetanus toxoid immunization among pregnant women in Ethiopia using data from Ethiopian demographic and health survey 2016

**DOI:** 10.1186/s12884-023-05911-z

**Published:** 2023-10-23

**Authors:** Beletech Fentie, Tewodros Getaneh Alemu, Masresha Asmare Techane, Chalachew Adugna Wubneh, Nega Tezera Assimamaw, Getaneh Mulualem Belay, Tadesse Tarik Tamir, Addis Bilal Muhye, Destaye Guadie Kassie, Amare Wondim, Bewuketu Terefe, Bethelihem Tigabu Tarekegn, Mohammed Seid Ali, Almaz Tefera Gonete, Berhan Tekeba, Selam Fisiha Kassa, Bogale Kassahun Desta, Amare Demsie Ayele, Melkamu Tilahun Dessie, Kendalem Asmare Atalell

**Affiliations:** 1 Department of Pediatrics and Child Health Nursing, School of Nursing, College of Medicine and Health Sciences, University of Gondar, Gondar, Ethiopia; 2 Department of Community Health Nursing, School of Nursing, College of Medicine and Health Sciences, University of Gondar, Gondar, Ethiopia

**Keywords:** Ethiopia, Immunization, Pregnant women, Spatial analysis, Tetanus Toxoid

## Abstract

**Introduction:**

Tetanus is a major public health problem caused by clostridium tetani. Although it is vaccine-preventable, the case fatality rate among neonates in areas with poor immunization coverage and limited access to clean deliveries reaches 80-100%. Vaccination of pregnant mothers with the tetanus toxoid (TT) vaccine is the most effective way to protect against neonatal tetanus. This study aimed to examine the spatial distribution and determinants of tetanus toxoid immunization among pregnant mothers using the 2016 EDHS data.

**Method:**

Secondary analysis of the Ethiopia Demographic and Health Survey 2016 was done to assess the spatial distribution and determinants of tetanus toxoid vaccine among pregnant women in Ethiopia. Spatial autocorrelation analysis and hot spot analysis were used to detect spatial dependency and spatial clustering of the tetanus toxoid vaccine in Ethiopia. Spatial interpolation was used to predict the tetanus toxoid vaccine coverage in unsampled areas. The multilevel binary logistic regression model was fitted to identify factors associated with tetanus toxoid vaccination. An adjusted odds ratio with 95% CI was calculated and used as the measure of association and a p-value less than 0.05 were considered statistically significant.

**Result:**

From the total of 7043 pregnant women, 42.4% of them have taken at least two doses of tetanus toxoid immunization. Spatial clustering of TT immunization was observed in the Northern, Southwestern and Southwestern parts of Ethiopia. Whereas, low TT coverage was observed in the Eastern and Western parts of the country. Increased ANC visits and the richest economic status favored TT immunization, whereas living in Addis Ababa and Dire Dewa cities decreased the TT immunization coverage.

**Conclusion:**

The finding of this study reveals that TT immunization had spatial dependency, with the highest immunization coverage observed in the Northern, Southwestern and Southeastern parts of the Country. Thus, geographically targeted interventions should be implemented particularly in the eastern and western parts of the country.

## Introduction

Tetanus is a vaccine-preventable disease caused by clostridium tetani. Vaccination of pregnant mothers with the Tetanus toxoid (TT) vaccine is one of the most effective ways to protect against neonatal tetanus disease [1, 2].

Maternal and neonatal tetanus (MNT) remains a major public health problem,with 80-100% case-fatality rate among neonates, especially in areas with poor immunization coverage and limited access to clean deliveries [3, 4]. Globally, an estimated 3.3 million neonatal death occur each year and about 9,000 babies died during the neonatal period every day. From this death, neonatal tetanus shares a high number (34,019 neonatal death) as WHO estimated in 2015 [5, 6].

One of the elimination strategies of neonatal tetanus is achieving ≥ 80% coverage with ≥ 2 doses of tetanus toxoid-containing vaccine (TTCV) among women of reproductive age through routine immunization of pregnant women and supplementary immunization activities [2, 7].

In many parts of low-income countries, however, the efforts made to improve maternal and neonatal protection through TT immunization programs with valid doses of TT (two and more) immunization coverage remains low [1, 3, 8].

In addition, a systemic review conducted in different countries attained at least 80% coverage for women of reproductive age receiving at least two doses of tetanus toxoid-containing vaccine or protection at birth [9].Barriers to maternal and neonatal tetanus elimination were mostly related to health systems and socioeconomic factors [5, 10].

In Ethiopia, the prevalence of tetanus toxoid injection is different in different regions [11]. The variation in the prevalence of TT immunization in pregnant mothers could provide a clue to identify high-risk areas by using spatial methods [11].

Different studies conducted on TT immunization revealed that socioeconomic, and health-related factors were associated with TT immunization [12–15]. Even though studies were conducted to identify individual and community-level factors, there was limited evidence on the spatial distribution of TT immunization among pregnant mothers in Ethiopia.

Therefore, this study aimed to determine the high-risk area (hotspot) of TT immunization among pregnant mothers through spatial analysis and identifying factors associated with it.

## Methods

### Study setting

This study was conducted in Ethiopia using the Ethiopian Demographic and Health Survey (EDHS) 2016. The 2016 EDHS is the fourth national representative cross-sectional survey, which is conducted by the Ethiopian Central Statistical Agency (CSA). The Ethiopian Public Health Institute (EPHI) surveyed in collaboration with the (CSA). The survey was conducted based on a nationally representative sample that provided estimates at the national and regional levels and for urban and rural areas as well.

Ethiopian 2016 demographic and health survey includes nine regions and two administrative cities in Ethiopia. It used sampling frame and multistage sampling technique. This survey includes a total of 645 selected enumeration areas. The survey collects data on the characteristics of the household and lists all household members including TT immunization of pregnant mothers. The target population is all pregnant women in Ethiopia.

### Study population

The study population was pregnant mothers in the randomly selected enumeration areas (EAs) of Ethiopia.

### Data source

Secondary data analysis from the 2016 Ethiopian demographic health survey was employed to identify the spatial distribution of TT immunization among pregnant mothers. An authorization letter for the use of this data was obtained from measure DHS and the data set was downloaded from the website; www.measuredhs.com. The survey covered all nine regions and two administrative cities in Ethiopia.

### Sample size determination and sampling procedure

**Fig. 1:**
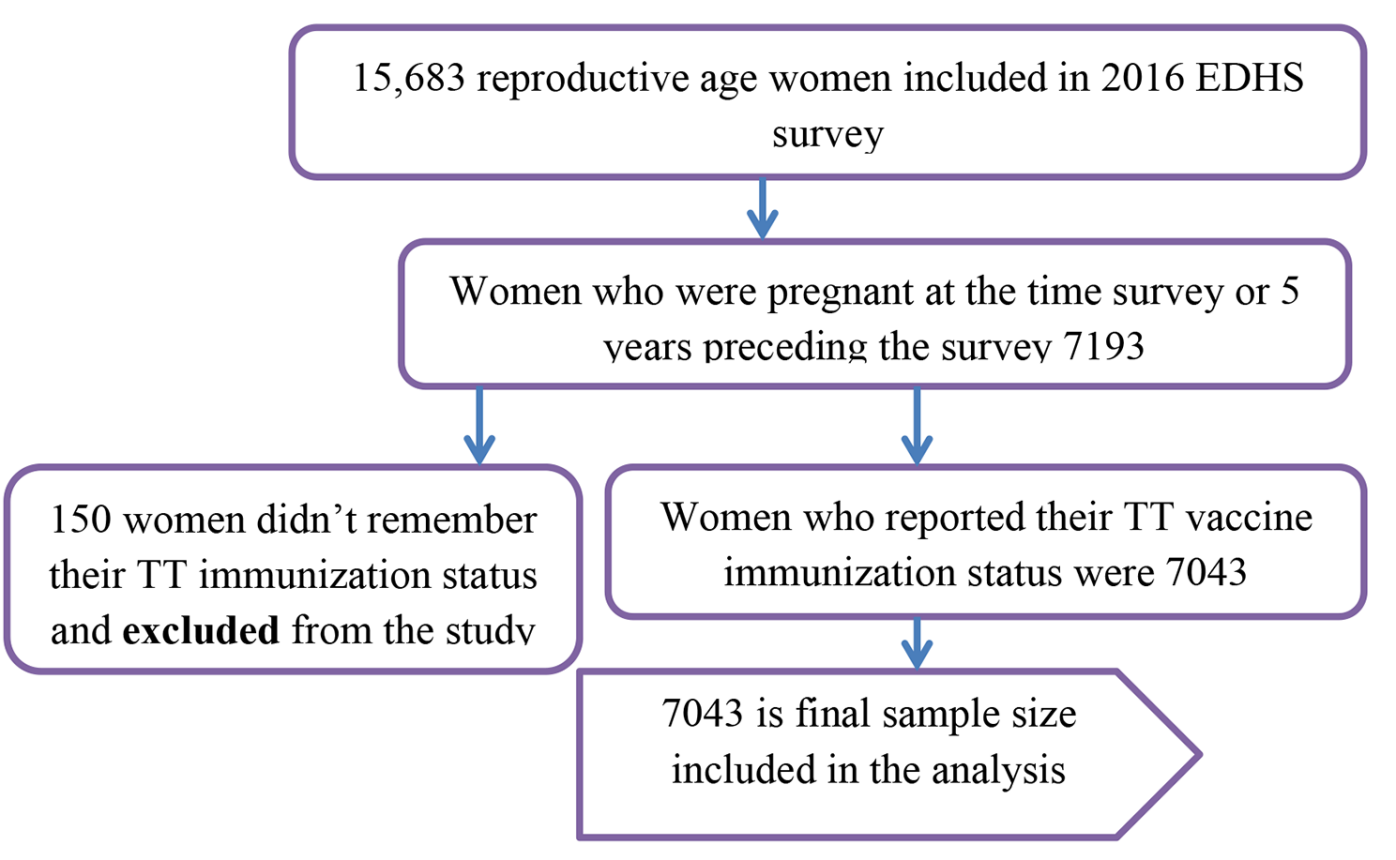
Diagrammatic representation of the sampling procedure

A two-stage stratified cluster sampling was employed to select study participants. From 645 enumeration areas, 15,683 reproductive-age women were included in the survey. About 7193 women were pregnant at the time of the survey or gave birth five years preceding the survey. But 150 women who did not remember TT immunization were excluded from the survey. Therefore the current study includes 7043 pregnant women who gave birth in five years preceding the survey (Fig. 1).

### Variables

#### Dependent variables

The main outcome variable of this study was TT immunization. Data about TT immunization were collected from mothers who were pregnant within 5 years before the survey either by accessing TT immunization through record review or by the mother’s report. Pregnant mothers who took 2 or more doses of the tetanus toxoid vaccine were classified as “protected ” and those who took less than two doses were classified as “ non-protected ” in the current study [1].

Mothers without a record of TT immunization were excluded from the study.

#### Independent variables

From the 2016 EDHS dataset maternal educational status(no education,primery,secondery and higher), ANC visit(no visit,< 3visit and ≥ 3 visits, residence(urban,rural), wealth index(poorest,poor,middle,richer and richest) and region were considered independent variables.

### Data management and analysis

Individual (women’s) record was used from the 2016 EDHS. For the spatial analysis, data management was done by using STATA version 14 software and Microsoft Excel 2007. Mapping was done using ArcGIS version 10.8.

A multilevel logistic regression model was the best-fitted model for the data after LR and ICC tests were checked. In this study, deviance was used for model comparison and the final model was the best-fitted model with the lowest deviance value. The ICC value was 0.258 in the null model, which indicates that about 25.8%% of the overall variability of TT immunization coverage was due to cluster variability. A two-level logistic regression model was fitted to identify factors associated with TT immunization coverage of pregnant mothers in Ethiopia. ANC visits, region and individual wealth index were statistically significant in the multivariable multilevel logistic regression.

### Spatial autocorrelations

Spatial autocorrelation (Global Moran’s I) was done to test for spatial clustering of tetanus toxoid immunization among pregnant mothers in Ethiopia. Moran’s I statistics is used to measure whether TT coverage in Ethiopia was distributed randomly, clustered, or dispersed, by taking the entire dataset and producing a single output value, which ranges from − 1 to 1. Moran’s I value close to -1 indicates TT immunization is dispersed in the area. On the other hand, Moran’s I value closer to 1 indicates TT immunization coverage is clustered in the area. Whereas 0 Moran’s I value means the data is randomly distributed. A statistically significant Moran’s I(p < 0.05) leads to the rejection of the null hypothesis (TT immunization is randomly distributed) and accepting the alternative hypothesis that indicates there is spatial dependence of TT immunization in Ethiopia.

### Hotspot analysis of TT immunization coverage

Hotspot analysis (Getis-Ord Gi*) was used to identify the spatial clustering of TT immunization in Ethiopia.

### Spatial interpolation

Spatial Kriging interpolation was used to estimate the distribution of TT immunization across the region.

### Ethical approval and consent to participate

Permission was obtained from measure demographic and health survey through an online request at http//www.dhsprogram.com and the information obtained was not disclosed to the third party.

All methods were performed following the relevant guidelines and regulations.

## Result

### Socio-demographic characteristics of the study population

In this study, 7043 pregnant mothers were involved. The majority (32.58%) of the study participants were Orthodox Tewahido religion followers whereas the least were traditional religion followers. Related to residency, three-fourths (79.24%) were rural dwellers. The highest number of participants were from the Oromia regional state(14.15%) and most (60.72%) were illiterate. Regarding maternal age, 28% were in the age groups of 25–29 years and 35.9% of mothers had ≥ 4 antenatal visits (Table 1).

**Table 1:** Socio demographic characteristics of study population in Ethiopia, EDHS 2019

Variables	Category	Frequency	Percent
Religion	Orthodox	2,295	32.58%
	Catholic	48	0.68%
	Protestant	1,320	18.74%
	Muslim	3,269	46.4%
	Traditional	63	0.89%
	Others	48	8.68%
Residence	Urban	1,462	20.75%
	Rural	5,581	79.24%
Region	Tigray	750	10.64%
	Afar	641	9.1%
	Amhara	741	10.52%
	Oromia	997	14.15%
	Somali	799	11.34%
	Benshangul	568	8.06%
	SNNPR	884	12.55%
	Gambela	526	7.46%
	Harari	406	5.76%
	Addis Ababa	363	5.15%
	Dire dawa	368	5.22%
Maternal Education	No education	4,277	60.72%
	Incomplete primary	1,729	24.54%
	Complete primary	170	2.41%
	Incomplete secondary	487	6.91%
	Complete secondary	78	1.1%
	Higher	302	4.28%
Maternal Age	15–19	354	5.02%
	20–24	1,473	20.9%
	25–29	1,973	28%
	30–34	1,489	21.1%
	35–39	1,116	15.8%
	40–44	472	6.7%
	45–49	166	2.35%
ANC Visits	No visits	2,454	34.8%
	< 3visits	2,058	29.22%
	≥ 3 visit	2,531	35.9%
Community level Literacy	Low	6,951	44.75%
	High	8,582	55.25%
Community level poverty	Low	7,821	50.35%
	High	7,712	49.65%

### Tetanus toxoid vaccine immunization status

**Fig. 2:**
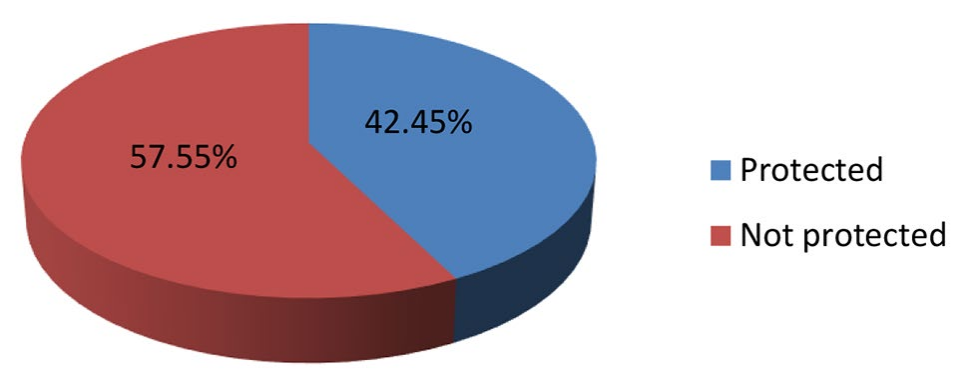
Distribution of tetanus vaccine among pregnant mothers in Ethiopia

Pregnant mothers who took a minimum of two doses of the tetanus toxoid vaccine were categorized as protected [16]. In the current study, about 42.4% of pregnant mothers have taken a minimum of two doses of the tetanus toxoid vaccine i.e. protected (Fig. 2). When it is seen in the regions of the country, the highest (65.76%) protected mothers were found in the Harari region whereas the minimum (23.56%) were found in the afar regional state of Ethiopia (Table 2).

**Table 2:** Tetanus vaccine coverage based on the regional states of Ethiopia

Region	Not protected	Protected
	N (%)	N (%)
Tigray	452(60.27)	298(39.73)
Afar	490(76.44)	151(23.56)
Amhara	476(64.24)	265(35.76)
Oromia	580(58.17)	417(41.83)
Somali	545(68.21)	254(31.79)
Benishangul	318(55.99)	250(44.01)
SNNPR	489(55.32)	395(44.68)
Gambela	298(56.65)	228(43.35)
Harari	139(34.24)	267(65.76)
Addis Ababa	134(36.91)	229(63.09)
Dire Dewa	132(35.87)	236(64.13)

### Spatial analysis of tetanus toxoid immunization

#### Spatial autocorrelation

**Fig. 3:**
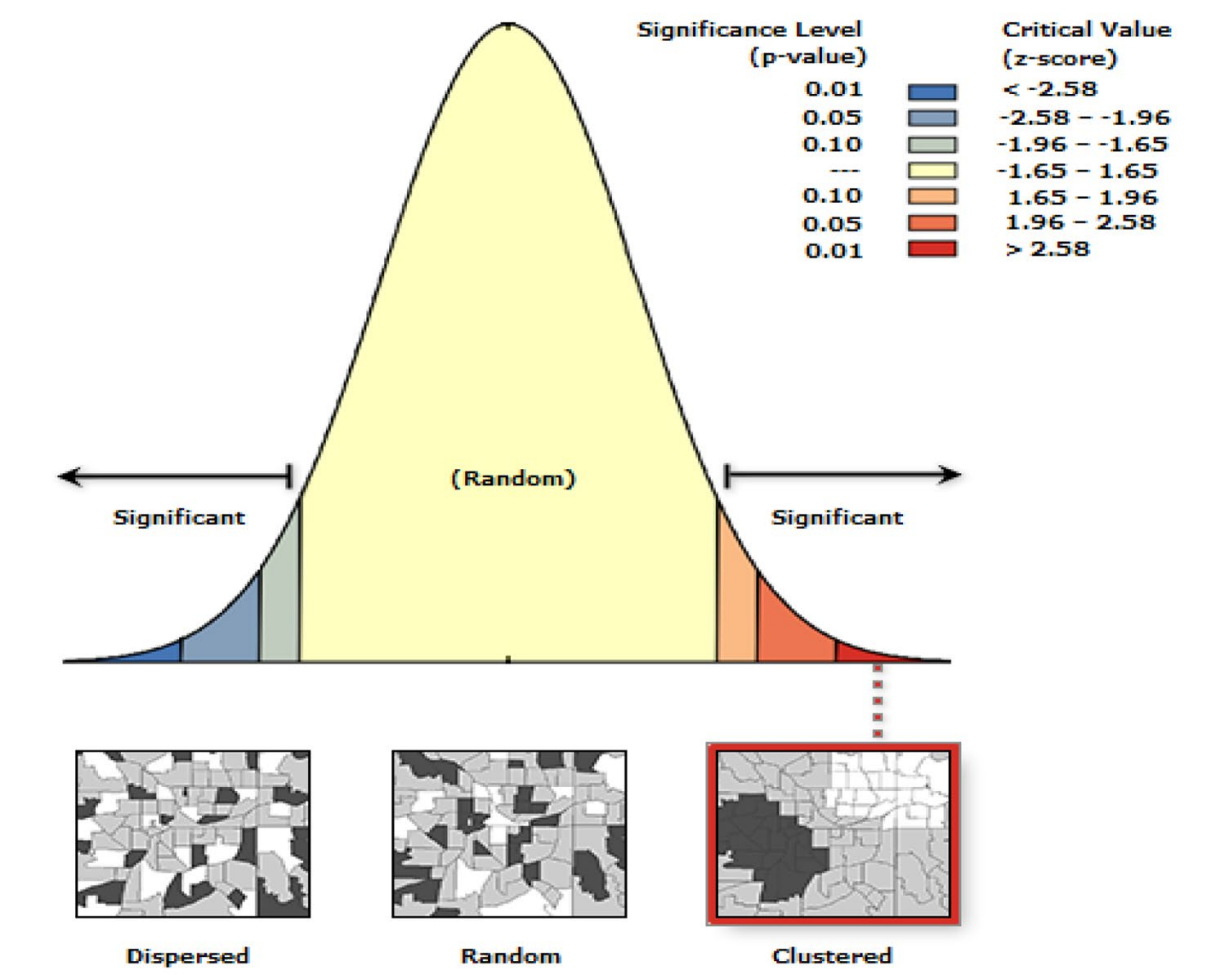
Spatial Autocorrelation Report of tetanus toxoid immunization coverage

The autocorrelation analysis of EDHS 2016 showed that the spatial distribution of tetanus toxoid immunization was significantly varied across Ethiopia, with a Global Moran’s I value of 0.242 (p-value < 0.0001) and a z-score of 15.16. This indicates TT immunization coverage in Ethiopia has spatial dependence (Fig. 3).

#### Hotspot analysis of TT immunization in Ethiopia

**Fig. 4:**
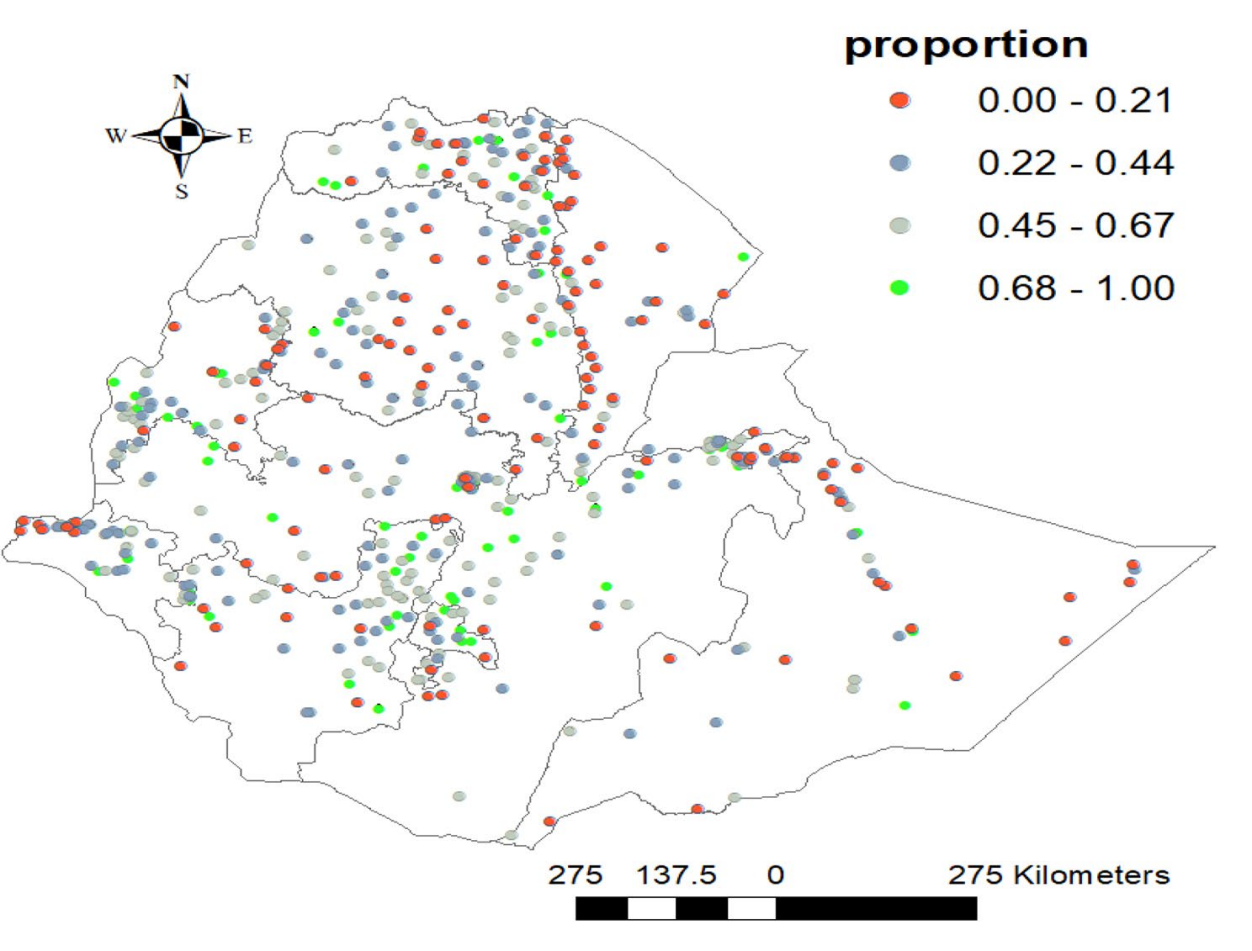
Hot spot analysis result of tetanus toxoid immunization coverage

In the mapping of TT immunization, significant spatial clusters were observed in the Northern, western and central parts of Ethiopia. TT immunization spatial dispersion was observed in the Northern, northeastern, and western parts of the country (Fig. 4).

#### Interpolation of TT immunization

**Fig. 5:**
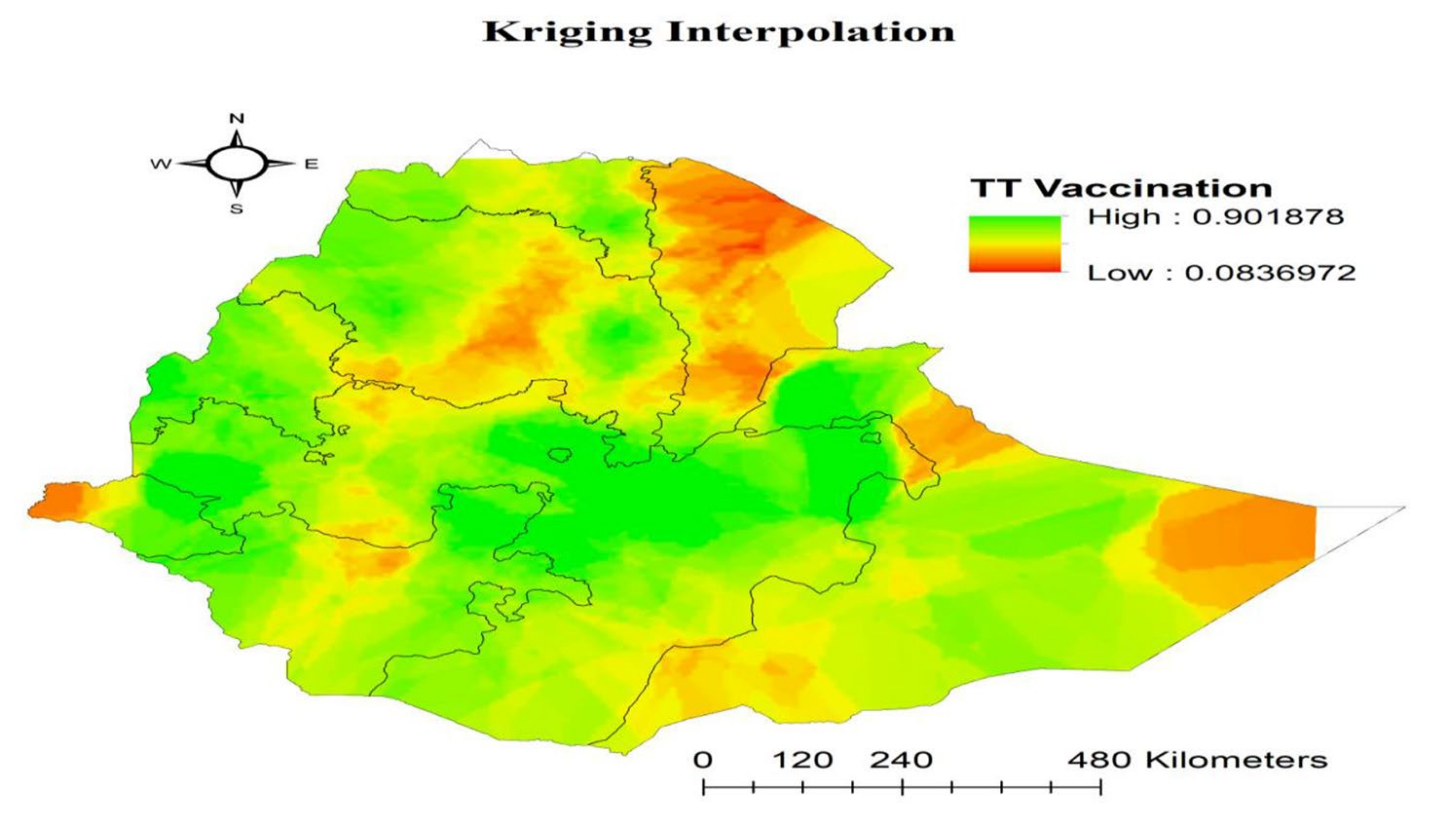
Kriging interpolation analysis result of tetanus toxoid immunization coverage

Kriging interpolation was used to map the predicted TT immunization coverage in the observed areas. The highest TT immunization coverage was observed in the Tigray, Amhara, Addis Ababa and Oromia. But, low TT vaccine coverage was observed in the Afar, Somali and Gambela (Fig. 5).

#### Determinants of Tetanus toxoid vaccine immunization in Ethiopia

A multilevel logistic regression model was the best-fitted model for the data after LR and ICC tests were checked. In this study, deviance was used for model comparison and the final model was the best-fitted model with the lowest deviance value. The ICC value was 0.258 in the null model, which indicates that about 25.8%% of the overall variability of TT immunization coverage was due to cluster variability.

Moreover, the PCV valve in the fnal model indicates the variation in the TT immunization coverage among study participants was clarified by both the individual and community level factors. Model ftness was done using loglikelihood and deviance test, then the final model (Model IV) has the highest loglikelihood and the lowest deviance and was taken as the best-ftted model.

Therefore number of ANC visits, region and individual wealth index were statistically significant in the multivariable multilevel logistic regression. The odds of protective TT immunization among pregnant mothers with no ANC visit and less than 3 visits were 48% [AOR = 0.52, 95%CI (0.43, 0.64)] and 41% [AOR = 0.59,95%CI(0.51, 0.68)] respectively compared to those with ANC visit of ≥ 3. Only 35% of mothers with the poorest income status took a protective dose of TT vaccine [AOR = 0.65, 95%CI (0.50, 0.86)] as compared to those with the richest income level. Based on the regions of the participants, 37% [AOR = 0.63, 95% CI (0.43, 0.81)] and 59% [AOR = 0.41, 95%(0.32, 0.65)] reside in Addis Ababa and Dire Dewa respectively took protective TT immunization (Table 3).

**Table 3:** Multilevel logistic regression analysis of both individual and community-level factors associated with TT immunization among pregnant mothers in Ethiopia

Individual and community-level characteristics		Model II AOR (95%CI)	Model III AOR (95%CI)	Model IV AOR (95%CI)
Place of residence	Urban		1	1
	Rural		0.55(0.42,0.72)	1.12(0.84,1.49)
ANC visits	no visits	0.59 (0.04, 0.07)		0.52(0.43,0.64)
	< 3visits	0.62(0.54,0 0.71)		0.59(0.51,0.68)
	≥ 3 visits	1		1
Maternal age	15–19	0.97(0.73,1.29)		0.95(0.71,1.26)
	20–24	0.98(0.82,1.17)		0.99(0.84,1.78)
	25–29	1		1
	30–34	1.08(0.92,1.29)		1.08(0.91,1.27)
	35–39	0.89(0.74,1.07)		0.91(0.75,1.09)
	40–44	0.85(0.65,1.12)		0.89(0.69,1.17)
	45–49	1.05(0.68,1.60)		1.09(0.72,1.66)
Maternal educational status	no education	0.79(0.58,1.09)		0.52 (0.35,1,02)
	primary	0.95(0.71,1.30)		0.96 (0.71,1.30)
	Secondary	0.87(0.63,1.22)		0.89 (0.64,1.23)
	higher	1		1
Wealth index	Poorest	0.59 (0.48, 0.73)		0.65(0.50,0.86)
	Poor	0.82(0.65, 1.02)		0.87(0.66,1.15)
	Middle	0.79(0.63,0.98)		0.83(0.63,1.09)
	Richer	1.04(0.83,1.30)		1.07(0.81,1.41)
	Richest	1		1
Region	Tigray		1	1
	Afar		0.56(0.37,0.85)	1.83(0.68, 4.94)
	Amhara		1.12(0.78,1.62)	1.58(0.61, 4.14)
	Oromia		1.38(0.97,1.96)	1.73(0.70 4.28)
	Somali		0.86(0.59,1.26)	2.78(1.05, 7.36)
	Benshangul		1.45(0.98,2.14)	2.17(0.86, 5.51)
	SNNPR		0.86(0.59,1.26)	0.91(0.33, 2.48)
	Gambela		1.07(0.72,1.58)	1.76(0.66, 4.71)
	Harari		3.19(2.06,4.94)	1.60(0.57, 4.46)
	Addis Ababa		1.29(0.82,2.01)	0.63(0.43, 0.81)
	Dire Dewa		2.29(1.47,3.56)	0.41(0.32, 0.65)
Community income level	Low		0.59(0.53,0.68)	0.81(0.59,1.11
	High		1	1
Community literacy level	Low		0.75(0.59,0.95)	0.94(0.76,1.14)
	High		1	1
ICC		12.8%	16.1%	7.9%
Log likelihood		-3717.269	-4333.5278	-3657.1076
Deviance		7434.538	8667.055	7314.2152

## Discussion

The overall TT vaccination coverage among pregnant women in Ethiopia was 42.4% which is lower than the study conducted at the University of Gondar Comprehensive Specialized Hospital, Ethiopia 69.8% [17], in Southern Ethiopia 51.8 [13], 72.5% [14], India 68% [2], Sudan 60% [18] and Sera Leon 82.12% [19]. The finding from this study is also far below the WHO recommendation, which states 80% immunization coverage to achieve neonatal tetanus elimination [7]. The lower immunization coverage in our study might be because this study is country-wide, which includes the very remote areas, where immunization services were low. On the other hand, the previous studies were conducted mainly in urban, where the immunizations services were relatively good [12, 20].

However, the current finding is higher than the study conducted in Turkey 27.8% [6]. The possible reason could be the sociocultural and economic differences between the two countries. This study is also lower than the study conducted in Dukem town Ethiopia. The higher immunization coverage in our study over the Dukem study could be because of the population difference. The Dukem study was conducted among all reproductive-age women, which might be linked with awareness. Pregnant women had ANC follow-ups, which might have a chance to have advice about the importance of TT immunization for them and their children.

In our study ANC follow-up, geographic region and individual level wealth status were statistically significant in the multivariable multilevel logistic regression model.

In agreement with previous studies conducted in Ethiopia, Sudan, Cameroon, Egypt and five African countries pregnant mothers who have ANC follow-up had 3 times more likely to be immunized for TT [6, 11, 21–23] as compared with pregnant mothers who had no ANC follow-up.

The poorest income level of the participants negatively affected TT vaccination in the current study as compared to the richest. This finding is in agreement with the studies conducted in different regions of the world [3, 24]. This finding also in line with the studies conducted in ten east African countries, Sudan and EDHS in five African countries that showed women who has high wealth index took protective dose of TT [18, 25, 26]. This could be due to economic constraints for transportation to health facilities and delay for ANC visits.

Regionally, participants who live in Addis Ababa and Dire Dewa didn’t get adequate TT immunization as compared to those live in Tigray. We expect dwellers of Addis Ababa would get adequate TT immunization as most health facilities are concentrated to this area, but it is not true in the current research finding.

### Strength and limitation of the study

The strength of the current study was based on EDHS national data, which is more representative for the total population in the nation and the model of data analysis was spatial and multilevel analysis. As a limitation, the data was only from EDHS 2016 data which doesn’t indicate the trend of TT immunization. In addition, the cross-sectional design of the 2016 EDHS data limits the ability to establish a causal relationship between the studied exposures and tetanus toxoid immunization.

## Conclusion

The finding of this study reveals that TT immunization had spatial dependency. Therefore policy makers could give attention for eastern and western part of the country which has low TT immunization. Additionally, lower ANC visit, poorest income level and living in Addis Ababa and Dire Dewa decreased the utilization of protective dose of TT immunization among pregnant mothers. Therefore, the health administrators of these two cities shall create awareness of mothers towards TT immunization using different mechanisms of communication like mass medias.

## Data Availability

All data are available in open access repository. The data are publicly available at DHS MEASURE website archive@measuredhs.com.

## Abbreviations

ANC: Ante Natal Care
AOR: Adjusted Odds Ratios
EAs: Enumeration Areas
CSA: Central Statistical Agency
EDHS: Ethiopian Demographic Health Survey
KDHS: Kenian Demographic Health Survey
TTCV: Tetanus Toxoid Containing Vaccine
TT: Tetanus Toxoid
